# Supplementary material for: Effects of weather and season on human brain volume
Source: PLoS One. 2021 Mar 24;16(3):e0236303. doi: 10.1371/journal.pone.0236303 (PMC7990212; doi:10.1371/journal.pone.0236303)
Supplement: S1 File — (DOCX) [file pone.0236303.s003.docx]

**Supplemental Tables**

| (a) **Age, by month** - FDR corrected | | | | | | | | | | | |
| --- | --- | --- | --- | --- | --- | --- | --- | --- | --- | --- | --- |
|  | Jan | Feb | Mar | Apr | May | Jun | Jul | Aug | Sep | Oct | Nov |
| Feb | 0.583 | - | - | - | - | - | - | - | - | - | - |
| Mar | **** 0.005** | *** 0.033** | - | - | - | - | - | - | - | - | - |
| Apr | 0.135 | 0.413 | 0.261 | - | - | - | - | - | - | - | - |
| May | 0.135 | 0.413 | 0.294 | 0.949 | - | - | - | - | - | - | - |
| Jun | 0.739 | 0.831 | *** 0.015** | 0.282 | 0.282 | - | - | - | - | - | - |
| Jul | 0.198 | 0.495 | 0.218 | 0.887 | 0.882 | 0.358 | - | - | - | - | - |
| Aug | 0.886 | 0.501 | **** 0.005** | 0.117 | 0.117 | 0.663 | 0.135 | - | - | - | - |
| Sep | 0.850 | 0.739 | *** 0.015** | 0.261 | 0.261 | 0.887 | 0.299 | 0.739 | - | - | - |
| Oct | *** 0.023** | 0.117 | 0.688 | 0.501 | 0.542 | 0.068 | 0.440 | *** 0.015** | 0.065 | - | - |
| Nov | 0.542 | 0.949 | *** 0.034** | 0.440 | 0.439 | 0.773 | 0.501 | 0.468 | 0.728 | 0.128 | - |
| Dec | 0.379 | 0.739 | 0.117 | 0.677 | 0.663 | 0.574 | 0.739 | 0.295 | 0.501 | 0.282 | 0.742 |
|  |  |  |  |  |  |  |  |  |  |  |  |
| (b) **Sex, by month** - FDR corrected | | | | | | | | | | | |
|  | Jan | Feb | Mar | Apr | May | Jun | Jul | Aug | Sep | Oct | Nov |
| Feb | 0.991 | - | - | - | - | - | - | - | - | - | - |
| Mar | 0.991 | 0.983 | - | - | - | - | - | - | - | - | - |
| Apr | 0.596 | 0.596 | 0.606 | - | - | - | - | - | - | - | - |
| May | 0.596 | 0.596 | 0.470 | 0.233 | - | - | - | - | - | - | - |
| Jun | 0.233 | 0.233 | 0.233 | 0.596 | 0.065 | - | - | - | - | - | - |
| Jul | 0.337 | 0.330 | 0.340 | 0.682 | 0.122 | 0.858 | - | - | - | - | - |
| Aug | 0.606 | 0.596 | 0.654 | 0.991 | 0.233 | 0.596 | 0.683 | - | - | - | - |
| Sep | 0.991 | 0.991 | 0.987 | 0.596 | 0.596 | 0.241 | 0.340 | 0.606 | - | - | - |
| Oct | 0.163 | 0.163 | 0.163 | 0.372 | *** 0.027** | 0.887 | 0.682 | 0.401 | 0.184 | - | - |
| Nov | 0.804 | 0.786 | 0.858 | 0.858 | 0.337 | 0.372 | 0.596 | 0.858 | 0.804 | 0.241 | - |
| Dec | 0.991 | 0.991 | 0.943 | 0.596 | 0.596 | 0.233 | 0.330 | 0.596 | 0.991 | 0.163 | 0.756 |
|  |  |  |  |  |  |  |  |  |  |  |  |
| (c) **Motion, by month** - FDR corrected | | | | | | | | | | | |
|  | Jan | Feb | Mar | Apr | May | Jun | Jul | Aug | Sep | Oct | Nov |
| Feb | 0.820 | - | - | - | - | - | - | - | - | - | - |
| Mar | 0.680 | 0.890 | - | - | - | - | - | - | - | - | - |
| Apr | 0.680 | 0.890 | 1.000 | - | - | - | - | - | - | - | - |
| May | 0.350 | 0.680 | 0.680 | 0.680 | - | - | - | - | - | - | - |
| Jun | 0.890 | 0.890 | 0.820 | 0.820 | 0.560 | - | - | - | - | - | - |
| Jul | 0.680 | 0.890 | 1.000 | 1.000 | 0.680 | 0.820 | - | - | - | - | - |
| Aug | 0.680 | 0.820 | 0.890 | 0.890 | 0.820 | 0.680 | 0.890 | - | - | - | - |
| Sep | 0.820 | 1.000 | 0.890 | 0.890 | 0.680 | 0.890 | 0.890 | 0.820 | - | - | - |
| Oct | 0.680 | 0.820 | 0.890 | 0.890 | 0.820 | 0.680 | 0.890 | 1.000 | 0.820 | - | - |
| Nov | 0.820 | 1.000 | 0.890 | 0.890 | 0.680 | 0.890 | 0.890 | 0.820 | 1.000 | 0.820 | - |
| Dec | 0.680 | 0.890 | 0.890 | 0.890 | 0.820 | 0.760 | 0.890 | 0.960 | 0.890 | 0.960 | 0.890 |
|  |  |  |  |  |  |  |  |  |  |  |  |
| (d) **Scan-sequence, by month** - FDR corrected | | | | | | | | | | | |
|  | Jan | Feb | Mar | Apr | May | Jun | Jul | Aug | Sep | Oct | Nov |
| Feb | 0.480 | - | - | - | - | - | - | - | - | - | - |
| Mar | 0.540 | 0.760 | - | - | - | - | - | - | - | - | - |
| Apr | 0.760 | 0.540 | 0.730 | - | - | - | - | - | - | - | - |
| May | 0.980 | 0.460 | 0.530 | 0.750 | - | - | - | - | - | - | - |
| Jun | 0.510 | 0.150 | 0.170 | 0.360 | 0.510 | - | - | - | - | - | - |
| Jul | 0.750 | 0.640 | 0.750 | 0.880 | 0.740 | 0.330 | - | - | - | - | - |
| Aug | 0.360 | 0.850 | 0.730 | 0.500 | 0.360 | 0.150 | 0.540 | - | - | - | - |
| Sep | 0.600 | 0.220 | 0.320 | 0.490 | 0.610 | 0.850 | 0.450 | 0.170 | - | - | - |
| Oct | 0.750 | 0.320 | 0.360 | 0.600 | 0.750 | 0.640 | 0.530 | 0.230 | 0.750 | - | - |
| Nov | 0.980 | 0.450 | 0.530 | 0.750 | 0.980 | 0.510 | 0.730 | 0.360 | 0.610 | 0.750 | - |
| Dec | 0.750 | 0.600 | 0.750 | 0.980 | 0.750 | 0.360 | 0.950 | 0.530 | 0.490 | 0.600 | 0.750 |

Supplement Table 1(a-d) – Paired t-tests by month for age, sex, motion, and scan sequence. FDR corrected. N=3279
* - p < 0.05
** - p < 0.01

|  |  |  | Scan type vs 1 | | |  |  |  | Deviation from normal | | | vs January | | | | | | | | | | |
| --- | --- | --- | --- | --- | --- | --- | --- | --- | --- | --- | --- | --- | --- | --- | --- | --- | --- | --- | --- | --- | --- | --- |
|  | (Intercept) | Sexcode | scan 3 | scan 5 | scan 6 | age | **BMI** | motion4 | Pressure | Temp | Humidity | Feb | Mar | Apr | May | Jun | Jul | Aug | Sep | Oct | Nov | Dec |
| BrainSegVol | 0.000 | 0.693 | 0.000 | 0.697 | 0.274 | 0.267 | **0.309** | 0.002 | 0.403 | 0.164 | 0.207 | 0.263 | 0.728 | 0.938 | 0.930 | 0.494 | 0.710 | 0.722 | 0.160 | 0.078 | 0.118 | 0.698 |
| TotalGrayVol | 0.000 | 0.092 | 0.000 | 0.830 | 0.503 | 0.000 | **0.257** | 0.000 | 0.405 | 0.108 | 0.110 | 0.545 | 0.990 | 0.846 | 0.630 | 0.669 | 0.633 | 0.585 | 0.325 | 0.087 | 0.288 | 0.743 |
| CerebralWhiteMatterVol | 0.000 | 0.238 | 0.113 | 0.367 | 0.126 | 0.000 | **0.697** | 0.088 | 0.419 | 0.545 | 0.520 | 0.105 | 0.396 | 0.417 | 0.587 | 0.398 | 0.841 | 0.938 | 0.132 | 0.148 | 0.043 | 0.563 |
| CortexVol | 0.000 | 0.265 | 0.004 | 0.676 | 0.637 | 0.000 | **0.328** | 0.000 | 0.167 | 0.205 | 0.042 | 0.446 | 0.804 | 0.750 | 0.535 | 0.721 | 0.618 | 0.658 | 0.537 | 0.201 | 0.261 | 0.640 |
| SubCortGrayVol | 0.000 | 0.000 | 0.003 | 0.123 | 0.813 | 0.001 | **0.901** | 0.049 | 0.908 | 0.403 | 0.736 | 0.805 | 0.899 | 0.928 | 0.965 | 0.696 | 0.246 | 0.166 | 0.487 | 0.203 | 0.882 | 0.840 |
| lhCerebralWhiteMatterVol | 0.000 | 0.248 | 0.062 | 0.389 | 0.111 | 0.000 | **0.637** | 0.062 | 0.596 | 0.438 | 0.558 | 0.106 | 0.371 | 0.430 | 0.645 | 0.414 | 0.928 | 0.948 | 0.133 | 0.161 | 0.031 | 0.618 |
| lhCortexVol | 0.000 | 0.239 | 0.002 | 0.756 | 0.484 | 0.000 | **0.322** | 0.000 | 0.281 | 0.138 | 0.047 | 0.450 | 0.832 | 0.717 | 0.447 | 0.722 | 0.746 | 0.652 | 0.517 | 0.219 | 0.235 | 0.751 |
| rhCerebralWhiteMatterVol | 0.000 | 0.233 | 0.199 | 0.351 | 0.146 | 0.000 | **0.762** | 0.125 | 0.282 | 0.668 | 0.489 | 0.109 | 0.427 | 0.410 | 0.536 | 0.387 | 0.756 | 0.928 | 0.135 | 0.140 | 0.061 | 0.514 |
| rhCortexVol | 0.000 | 0.303 | 0.011 | 0.607 | 0.811 | 0.000 | **0.345** | 0.000 | 0.100 | 0.303 | 0.042 | 0.452 | 0.781 | 0.787 | 0.637 | 0.727 | 0.511 | 0.671 | 0.566 | 0.193 | 0.299 | 0.546 |
| Left.Lateral.Ventricle | 0.000 | 0.024 | 0.813 | 0.186 | 0.282 | 0.000 | **0.363** | 0.771 | 0.326 | 0.339 | 0.564 | 0.538 | 0.318 | 0.149 | 0.117 | 0.973 | 0.596 | 0.443 | 0.995 | 0.430 | 0.331 | 0.607 |
| Right.Lateral.Ventricle | 0.000 | 0.075 | 0.395 | 0.160 | 0.276 | 0.000 | **0.398** | 0.515 | 0.985 | 0.616 | 0.905 | 0.209 | 0.126 | 0.051 | 0.117 | 0.665 | 0.903 | 0.426 | 0.616 | 0.264 | 0.285 | 0.379 |
| SupraTentorialVol | 0.000 | 0.972 | 0.040 | 0.324 | 0.335 | 0.109 | **0.421** | 0.004 | 0.257 | 0.262 | 0.161 | 0.239 | 0.830 | 0.930 | 0.867 | 0.510 | 0.668 | 0.732 | 0.244 | 0.148 | 0.111 | 0.643 |
| Brain.Stem | 0.000 | 0.965 | 0.000 | 0.083 | 0.371 | 0.000 | **0.202** | 0.035 | 0.369 | 0.239 | 0.395 | 0.951 | 0.514 | 0.687 | 0.674 | 0.873 | 0.615 | 0.782 | 0.259 | 0.268 | 0.988 | 0.587 |
| Left.Cerebellum.Cortex | 0.000 | 0.066 | 0.000 | 0.007 | 0.244 | 0.025 | **0.159** | 0.003 | 0.364 | 0.061 | 0.999 | 0.670 | 0.380 | 0.615 | 0.848 | 0.542 | 0.563 | 0.913 | 0.084 | 0.021 | 0.303 | 0.951 |
| Left.Cerebellum.White.Matter | 0.000 | 0.001 | 0.000 | 0.221 | 0.338 | 0.482 | **0.215** | 0.374 | 0.743 | 0.678 | 0.572 | 0.261 | 0.176 | 0.917 | 0.076 | 0.221 | 0.910 | 0.352 | 0.018 | 0.003 | 0.217 | 0.851 |
| Right.Cerebellum.Cortex | 0.000 | 0.205 | 0.000 | 0.012 | 0.325 | 0.007 | **0.154** | 0.002 | 0.267 | 0.043 | 0.874 | 0.796 | 0.638 | 0.928 | 0.791 | 0.927 | 0.727 | 0.626 | 0.196 | 0.069 | 0.855 | 0.919 |
| Right.Cerebellum.White.Matter | 0.000 | 0.000 | 0.000 | 0.193 | 0.184 | 0.749 | **0.321** | 0.585 | 0.539 | 0.102 | 0.995 | 0.727 | 0.715 | 0.220 | 0.433 | 0.965 | 0.712 | 0.400 | 0.074 | 0.102 | 0.772 | 0.373 |
| Left.Amygdala | 0.000 | 0.119 | 0.790 | 0.713 | 0.003 | 0.918 | **0.384** | 0.038 | 0.189 | 0.342 | 0.944 | 0.672 | 0.506 | 0.557 | 0.239 | 0.168 | 0.528 | 0.292 | 0.051 | 0.300 | 0.096 | 0.261 |
| Left.Caudate | 0.000 | 0.000 | 0.809 | 0.569 | 0.456 | 0.001 | **0.306** | 0.075 | 0.161 | 0.893 | 0.474 | 0.484 | 0.422 | 0.343 | 0.946 | 0.569 | 0.896 | 0.210 | 0.112 | 0.282 | 0.974 | 0.775 |
| Left.Hippocampus | 0.000 | 0.016 | 0.754 | 0.687 | 0.626 | 0.203 | **0.506** | 0.109 | 0.376 | 0.208 | 0.565 | 0.363 | 0.861 | 0.928 | 0.562 | 0.643 | 0.332 | 0.402 | 0.433 | 0.412 | 0.709 | 0.865 |
| Left.Pallidum | 0.000 | 0.005 | 0.000 | 0.704 | 0.310 | 0.144 | **0.891** | 0.545 | 0.820 | 0.935 | 0.826 | 0.491 | 0.835 | 0.742 | 0.814 | 0.678 | 0.462 | 0.006 | 0.155 | 0.725 | 0.805 | 0.723 |
| Left.Putamen | 0.000 | 0.102 | 0.299 | 0.298 | 0.435 | 0.000 | **0.988** | 0.127 | 0.249 | 0.206 | 0.439 | 0.933 | 0.847 | 0.766 | 0.866 | 0.901 | 0.482 | 0.041 | 0.375 | 0.579 | 0.688 | 0.822 |
| Left.Thalamus.Proper | 0.000 | 0.002 | 0.000 | 0.151 | 0.225 | 0.000 | **0.869** | 0.455 | 0.844 | 0.675 | 0.866 | 0.996 | 0.723 | 0.740 | 0.582 | 0.786 | 0.315 | 0.484 | 0.950 | 0.253 | 0.675 | 0.235 |
| Right.Amygdala | 0.000 | 0.289 | 0.833 | 0.765 | 0.031 | 0.158 | **0.708** | 0.046 | 0.376 | 0.286 | 0.488 | 0.698 | 0.969 | 0.637 | 0.604 | 0.245 | 0.369 | 0.437 | 0.413 | 0.284 | 0.262 | 0.405 |
| Right.Caudate | 0.000 | 0.000 | 0.849 | 0.348 | 0.290 | 0.000 | **0.193** | 0.052 | 0.482 | 0.913 | 0.463 | 0.265 | 0.462 | 0.273 | 0.446 | 0.223 | 0.813 | 0.832 | 0.056 | 0.298 | 0.616 | 0.229 |
| Right.Hippocampus | 0.000 | 0.009 | 0.348 | 0.490 | 0.352 | 0.300 | **0.983** | 0.005 | 0.231 | 0.404 | 0.771 | 0.674 | 0.600 | 0.558 | 0.717 | 0.177 | 0.479 | 0.419 | 0.238 | 0.084 | 0.613 | 0.431 |
| Right.Pallidum | 0.000 | 0.003 | 0.003 | 0.579 | 0.322 | 0.173 | **0.787** | 0.210 | 0.725 | 0.844 | 0.469 | 0.721 | 0.969 | 0.408 | 0.596 | 0.959 | 0.499 | 0.012 | 0.418 | 0.647 | 0.755 | 0.733 |
| Right.Putamen | 0.000 | 0.510 | 0.132 | 0.192 | 0.381 | 0.000 | **0.775** | 0.047 | 0.158 | 0.931 | 0.199 | 0.837 | 0.684 | 0.649 | 0.987 | 0.227 | 0.732 | 0.146 | 0.384 | 0.205 | 0.850 | 0.685 |
| Right.Thalamus.Proper | 0.000 | 0.000 | 0.000 | 0.033 | 0.050 | 0.000 | **0.950** | 0.303 | 0.931 | 0.425 | 0.933 | 0.711 | 0.747 | 0.408 | 0.785 | 0.557 | 0.122 | 0.262 | 0.489 | 0.304 | 0.440 | 0.225 |

Supplement table 2 – Linear regression of brain volumes vs variables (weather deviation from normal [weather effects], and month vs January [seasonal effects]) **including BMI**. N=517, p-values are uncorrected, red is p < 0.05. Skyra MRI scanner data only.

|  |  |  | **by Season** | | **Spring vs Summer** | | | **Summer vs Fall** | | | **Fall vs Winter** | | | **Winter vs Spring** | | | **Summer vs Winter** | | | **Spring vs Fall** | | |
| --- | --- | --- | --- | --- | --- | --- | --- | --- | --- | --- | --- | --- | --- | --- | --- | --- | --- | --- | --- | --- | --- | --- |
|  | **Df** | **N** | **F** | **p** | **p** | **Cohen's d** | **% change** | **p** | **Cohen's d** | **% change** | **p** | **Cohen's d** | **% change** | **p** | **Cohen's d** | **% change** | **p** | **Cohen's d** | **% change** | **p** | **Cohen's d** | **% change** |
| BrainSegVol | 506 | 517 | 1.586 | 0.192 | 0.414 | 0.033 | 0.27% | 0.364 | 0.041 | 0.34% | 0.447 | 0.026 | 0.22% | 0.023 | 0.100 | 0.83% | 0.121 | 0.066 | 0.56% | 0.086 | 0.076 | 0.61% |
| TotalGrayVol | 506 | 517 | 1.601 | 0.188 | 0.890 | -0.005 | -0.05% | 0.727 | 0.025 | 0.25% | 0.727 | 0.018 | 0.18% | 0.727 | 0.038 | 0.38% | 0.727 | 0.044 | 0.43% | 0.727 | 0.020 | 0.20% |
| CerebralWhiteMatterVol | 506 | 517 | 1.169 | 0.321 | 0.294 | 0.042 | 0.48% | 0.030 | 0.082 | 0.96% | 0.668 | 0.014 | 0.17% | 0.000 | 0.136 | 1.63% | 0.015 | 0.093 | 1.14% | 0.001 | 0.127 | 1.45% |
| CortexVol | 506 | 517 | 1.279 | 0.281 | 0.519 | -0.025 | -0.27% | 0.068 | 0.077 | 0.81% | 0.519 | 0.022 | 0.23% | 0.068 | 0.074 | 0.78% | 0.035 | 0.100 | 1.05% | 0.194 | 0.051 | 0.54% |
| SubCortGrayVol | 506 | 517 | 2.310 | 0.076 | 0.564 | -0.021 | -0.22% | 0.003 | 0.110 | 1.21% | 0.310 | 0.039 | 0.43% | 0.001 | 0.130 | 1.42% | 0.000 | 0.144 | 1.65% | 0.012 | 0.094 | 0.99% |
| lhCerebralWhiteMatterVol | 506 | 517 | 1.204 | 0.308 | 0.219 | 0.048 | 0.56% | 0.033 | 0.081 | 0.95% | 0.788 | 0.009 | 0.11% | 0.001 | 0.135 | 1.63% | 0.025 | 0.086 | 1.06% | 0.001 | 0.132 | 1.52% |
| lhCortexVol | 506 | 517 | 1.302 | 0.273 | 0.589 | -0.023 | -0.24% | 0.076 | 0.077 | 0.81% | 0.589 | 0.019 | 0.20% | 0.076 | 0.072 | 0.77% | 0.049 | 0.097 | 1.01% | 0.174 | 0.053 | 0.57% |
| rhCerebralWhiteMatterVol | 506 | 517 | 1.119 | 0.341 | 0.392 | 0.035 | 0.41% | 0.029 | 0.083 | 0.97% | 0.559 | 0.020 | 0.24% | 0.001 | 0.136 | 1.63% | 0.009 | 0.098 | 1.21% | 0.002 | 0.122 | 1.39% |
| rhCortexVol | 506 | 517 | 1.259 | 0.288 | 0.459 | -0.027 | -0.29% | 0.065 | 0.076 | 0.81% | 0.459 | 0.026 | 0.27% | 0.065 | 0.075 | 0.79% | 0.028 | 0.103 | 1.08% | 0.225 | 0.049 | 0.52% |
| Left.Lateral.Ventricle | 506 | 517 | 1.709 | 0.164 | 0.184 | 0.062 | 5.11% | 0.055 | -0.091 | -6.76% | 0.311 | 0.047 | 3.47% | 0.603 | 0.018 | 1.41% | 0.311 | -0.047 | -3.53% | 0.539 | -0.026 | -2.00% |
| Right.Lateral.Ventricle | 506 | 517 | 2.595 | 0.052 | 0.119 | 0.069 | 5.45% | 0.119 | -0.078 | -5.62% | 0.341 | 0.050 | 3.60% | 0.353 | 0.041 | 3.11% | 0.463 | -0.030 | -2.22% | 0.853 | -0.007 | -0.47% |
| SupraTentorialVol | 506 | 517 | 1.413 | 0.238 | 0.611 | 0.018 | 0.15% | 0.012 | 0.094 | 0.79% | 0.423 | 0.032 | 0.27% | 0.000 | 0.142 | 1.22% | 0.001 | 0.123 | 1.07% | 0.002 | 0.114 | 0.95% |
| Brain Stem | 506 | 517 | 0.181 | 0.909 | 0.206 | 0.050 | 0.74% | 0.010 | -0.110 | -1.67% | 0.206 | 0.046 | 0.73% | 0.673 | -0.015 | -0.22% | 0.159 | -0.061 | -0.95% | 0.159 | -0.064 | -0.95% |
| L Cerebellum Cortex | 506 | 517 | 2.187 | 0.089 | 0.206 | 0.051 | 1.17% | 0.001 | -0.131 | -3.06% | 0.523 | -0.021 | -0.55% | 0.007 | -0.099 | -2.46% | 0.000 | -0.153 | -3.59% | 0.030 | -0.078 | -1.92% |
| L Cerebellum WM | 506 | 517 | 1.669 | 0.173 | 0.277 | 0.045 | 1.46% | 0.052 | -0.086 | -2.81% | 0.400 | -0.028 | -1.01% | 0.089 | -0.068 | -2.39% | 0.010 | -0.114 | -3.79% | 0.277 | -0.041 | -1.39% |
| R Cerebellum Cortex | 506 | 517 | 1.474 | 0.221 | 0.311 | 0.042 | 0.95% | 0.004 | -0.118 | -2.69% | 0.933 | -0.003 | -0.07% | 0.044 | -0.075 | -1.84% | 0.004 | -0.120 | -2.76% | 0.044 | -0.073 | -1.77% |
| R Cerebellum WM | 506 | 517 | 0.991 | 0.397 | 0.185 | 0.053 | 1.76% | 0.010 | -0.114 | -3.80% | 0.814 | 0.008 | 0.29% | 0.185 | -0.050 | -1.82% | 0.014 | -0.101 | -3.52% | 0.156 | -0.060 | -2.10% |
| L Amygdala | 506 | 517 | 2.615 | 0.051 | 0.498 | 0.029 | 0.49% | 0.472 | 0.036 | 0.60% | 0.884 | 0.005 | 0.09% | 0.179 | 0.068 | 1.18% | 0.472 | 0.040 | 0.69% | 0.179 | 0.065 | 1.09% |
| L Caudate | 506 | 517 | 1.446 | 0.229 | 0.898 | -0.005 | -0.08% | 0.043 | 0.080 | 1.41% | 0.208 | 0.047 | 0.84% | 0.001 | 0.120 | 2.18% | 0.001 | 0.125 | 2.26% | 0.043 | 0.075 | 1.33% |
| L Hippocampus | 506 | 517 | 2.530 | 0.056 | 0.952 | -0.002 | -0.03% | 0.165 | 0.067 | 0.97% | 0.631 | -0.022 | -0.31% | 0.322 | 0.044 | 0.62% | 0.322 | 0.045 | 0.65% | 0.165 | 0.066 | 0.94% |
| L Pallidum | 506 | 517 | 1.258 | 0.288 | 0.462 | -0.026 | -0.41% | 0.217 | 0.049 | 0.80% | 0.094 | 0.070 | 1.07% | 0.021 | 0.099 | 1.46% | 0.005 | 0.114 | 1.88% | 0.462 | 0.026 | 0.39% |
| L Putamen | 506 | 517 | 3.401 | 0.018 | 0.068 | -0.069 | -1.22% | 0.000 | 0.130 | 2.32% | 0.200 | 0.045 | 0.76% | 0.004 | 0.109 | 1.84% | 0.000 | 0.170 | 3.09% | 0.080 | 0.065 | 1.07% |
| L Thalamus Proper | 506 | 517 | 0.644 | 0.587 | 0.659 | -0.016 | -0.21% | 0.029 | 0.087 | 1.18% | 0.470 | 0.030 | 0.40% | 0.012 | 0.099 | 1.37% | 0.008 | 0.113 | 1.58% | 0.057 | 0.072 | 0.96% |
| R Amygdala | 506 | 517 | 2.385 | 0.068 | 0.342 | 0.038 | 0.60% | 0.241 | 0.052 | 0.81% | 0.983 | -0.001 | -0.01% | 0.035 | 0.087 | 1.40% | 0.241 | 0.049 | 0.80% | 0.035 | 0.091 | 1.41% |
| R Caudate | 506 | 517 | 1.000 | 0.392 | 0.754 | -0.011 | -0.19% | 0.157 | 0.061 | 1.09% | 0.246 | 0.044 | 0.77% | 0.020 | 0.095 | 1.67% | 0.020 | 0.106 | 1.86% | 0.203 | 0.051 | 0.89% |
| R Hippocampus | 506 | 517 | 3.602 | 0.013 | 0.906 | -0.011 | -0.16% | 0.453 | 0.045 | 0.66% | 0.982 | 0.001 | 0.01% | 0.453 | 0.037 | 0.51% | 0.453 | 0.046 | 0.67% | 0.453 | 0.036 | 0.50% |
| R Pallidum | 506 | 517 | 1.140 | 0.332 | 0.299 | -0.039 | -0.65% | 0.099 | 0.061 | 1.04% | 0.099 | 0.069 | 1.08% | 0.026 | 0.096 | 1.47% | 0.001 | 0.127 | 2.14% | 0.480 | 0.025 | 0.39% |
| R Putamen | 506 | 517 | 2.529 | 0.057 | 0.022 | -0.085 | -1.48% | 0.000 | 0.136 | 2.47% | 0.231 | 0.042 | 0.71% | 0.011 | 0.099 | 1.67% | 0.000 | 0.176 | 3.20% | 0.129 | 0.056 | 0.95% |
| R Thalamus Proper | 506 | 517 | 1.119 | 0.341 | 0.417 | -0.029 | -0.38% | 0.024 | 0.089 | 1.17% | 0.121 | 0.055 | 0.75% | 0.002 | 0.116 | 1.54% | 0.000 | 0.138 | 1.92% | 0.116 | 0.063 | 0.79% |

Supplement Table 3 – ANOVA by season, **with BMI as a covariate**. N = 517. P-values are uncorrected.

|  | **Scan 4** | **Scan 5** | **Scan 6** | **Age** | **motion** | **pressure** | **temp** | **humidity** | **timeofday** | **Feb** | **Mar** | **Apr** | **May** | **Jun** | **Jul** | **Aug** | **Sep** | **Oct** | **Nov** | **Dec** |
| --- | --- | --- | --- | --- | --- | --- | --- | --- | --- | --- | --- | --- | --- | --- | --- | --- | --- | --- | --- | --- |
| **BrainSegVol** | 4.133 | 5.866 | 2.220 | -12.198 | -7.495 | 1.047 | -0.360 | 0.461 | -1.756 | -0.388 | 0.063 | ** -2.995 | -1.331 | -1.570 | -1.563 | -0.901 | -0.259 | -0.850 | * -2.241 | -1.244 |
| **TotalGrayVol** | 5.347 | 5.085 | 3.733 | -32.600 | -8.264 | 0.130 | -0.405 | 0.522 | -1.895 | 0.198 | 1.282 | -1.473 | 0.759 | -0.172 | -0.235 | 0.316 | 1.058 | 0.401 | -1.595 | -0.838 |
| **CerebralWhiteMatterVol** | -1.335 | 0.982 | -2.217 | 11.296 | -2.299 | 1.576 | -0.343 | 0.371 | -0.229 | -0.554 | -1.106 | *** -3.402 | ** -2.847 | ** -2.787 | -2.456 | -1.935 | -1.450 | -1.341 | -1.451 | -0.853 |
| **CortexVol** | 0.099 | -2.113 | -1.454 | -29.982 | -7.034 | 1.159 | -0.544 | 0.269 | -1.718 | 0.552 | 1.304 | -1.618 | 0.323 | -1.039 | -0.818 | -0.320 | 0.306 | 0.334 | -1.417 | 0.099 |
| **SubCortGrayVol** | -2.506 | -4.476 | -3.458 | -15.797 | 0.405 | 1.405 | 1.151 | -0.170 | -0.202 | -1.293 | 0.211 | *** -3.997 | * -2.288 | *** -3.874 | -2.321 | ** -3.041 | -1.571 | * -2.339 | * -2.170 | -1.207 |
| **lhCerebralWhiteMatterVol** | -1.404 | 0.913 | -2.426 | 11.077 | -2.156 | 1.595 | -0.291 | 0.460 | -0.341 | -0.648 | -1.104 | *** -3.391 | ** -2.810 | ** -2.703 | -2.365 | -1.907 | -1.294 | -1.264 | -1.418 | -0.713 |
| **lhCortexVol** | 0.172 | -1.784 | -1.560 | -30.349 | -6.951 | 1.122 | -0.397 | 0.449 | -1.690 | 0.358 | 1.281 | -1.677 | 0.312 | -0.976 | -0.961 | -0.404 | 0.389 | 0.315 | -1.514 | 0.133 |
| **rhCerebralWhiteMatterVol** | -1.251 | 1.041 | -1.984 | 11.397 | -2.418 | 1.541 | -0.391 | 0.279 | -0.115 | -0.454 | -1.097 | *** -3.377 | ** -2.854 | ** -2.843 | -2.521 | -1.942 | -1.591 | -1.403 | -1.469 | -0.984 |
| **rhCortexVol** | 0.025 | -2.401 | -1.322 | -29.089 | -6.991 | 1.175 | -0.680 | 0.087 | -1.714 | 0.734 | 1.305 | -1.531 | 0.328 | -1.083 | -0.661 | -0.230 | 0.218 | 0.347 | -1.295 | 0.063 |
| **Left.Lateral.Ventricle** | -0.103 | -1.115 | -1.363 | 19.317 | -1.159 | 1.636 | 0.654 | -1.497 | * -2.104 | -0.861 | -0.651 | -0.088 | -1.606 | -0.022 | 0.005 | -1.027 | -1.649 | -1.706 | * -2.065 | -0.307 |
| **Right.Lateral.Ventricle** | -1.225 | -1.771 | -1.869 | 19.597 | -1.131 | 1.612 | 0.990 | -1.680 | -1.797 | -1.745 | -1.588 | -0.779 | ** -2.681 | -1.229 | -0.843 | -1.773 | ** -2.609 | * -2.308 | * -2.216 | -1.089 |
| **SupraTentorialVol** | -0.970 | -0.936 | -2.700 | -7.739 | -5.539 | * 2.017 | -0.305 | 0.130 | -1.405 | -0.309 | -0.124 | *** -3.459 | * -2.130 | ** -2.739 | -2.305 | -1.848 | -1.179 | -1.130 | * -2.214 | -0.707 |
| **Brain.Stem** | 7.686 | 9.847 | 7.506 | 1.344 | -3.826 | -1.634 | 0.443 | 1.159 | -0.931 | -0.660 | 0.230 | -0.513 | -0.020 | 0.284 | -0.259 | 0.304 | 0.162 | -0.718 | -1.341 | -1.700 |
| **L Cerebellum.Cortex** | 12.580 | 16.480 | 12.249 | -14.925 | -5.736 | * -1.962 | -0.254 | 0.642 | -1.188 | -0.329 | 0.331 | 0.880 | 1.762 | * 2.463 | 1.803 | * 2.387 | * 2.326 | 1.088 | -0.422 | -1.875 |
| **L Cerebellum.White.Matter** | 11.377 | 16.306 | 9.937 | -6.484 | -4.945 | -1.439 | -0.369 | 0.761 | -1.003 | -0.294 | 0.150 | 0.754 | 1.422 | * 2.523 | 1.091 | * 2.142 | 1.894 | 0.045 | 0.245 | -0.582 |
| **R Cerebellum.Cortex** | 11.936 | 15.709 | 11.617 | -14.868 | -5.826 | * -2.387 | -0.048 | 0.795 | -0.971 | -0.235 | 0.577 | 0.386 | 1.570 | * 2.312 | 1.398 | 1.644 | * 2.035 | 0.564 | -0.458 | -1.448 |
| **R Cerebellum.White.Matter** | 12.081 | 16.935 | 10.652 | -6.132 | -5.084 | * -2.345 | 0.025 | 1.479 | -0.814 | 0.031 | 0.485 | 0.444 | 1.774 | ** 2.603 | 1.061 | * 2.148 | 1.536 | -0.254 | -0.224 | -0.912 |
| **L Amygdala** | -0.231 | -0.863 | -1.621 | -2.633 | -2.959 | -0.620 | 0.653 | 0.026 | -0.287 | * -2.466 | -0.829 | -1.572 | * -2.562 | * -2.166 | -1.100 | -0.353 | -1.388 | -1.226 | * -2.534 | -1.069 |
| **L Caudate** | -2.191 | -4.439 | -2.830 | -16.506 | -1.004 | * 2.281 | -0.681 | -1.339 | -1.426 | -0.941 | -0.155 | *** -3.871 | ** -2.586 | *** -3.466 | -1.231 | ** -2.776 | -1.563 | * -2.103 | * -2.454 | -1.513 |
| **L Hippocampus** | -1.577 | -0.718 | -2.504 | -3.911 | -0.842 | 0.776 | 0.851 | 0.798 | 0.348 | -0.869 | 0.008 | * -2.285 | -0.808 | -1.848 | -0.785 | -0.932 | 0.256 | -0.362 | -0.542 | -0.680 |
| **L Pallidum** | -2.036 | -3.405 | -1.452 | 3.670 | 0.792 | 1.010 | 0.887 | -0.256 | -1.414 | -0.640 | -0.497 | * -2.268 | -1.730 | *** -4.122 | -2.098 | * -2.489 | -1.523 | * -2.323 | ** -2.859 | 0.265 |
| **L Putamen** | 0.884 | -1.582 | 0.304 | -17.118 | 1.356 | 0.516 | 0.506 | -0.656 | 0.155 | -0.719 | 0.087 | ** -3.048 | -1.493 | *** -3.381 | ** -2.580 | ** -2.760 | -1.606 | -1.825 | -1.095 | -1.257 |
| **L Thalamus.Proper** | -2.056 | -3.391 | -5.233 | -15.493 | 0.559 | 0.684 | 0.849 | 0.233 | 0.672 | -1.618 | 0.129 | *** -3.310 | -1.797 | -1.843 | -1.976 | * -2.552 | -1.553 | -1.609 | -1.385 | -1.074 |
| **R Amygdala** | 0.305 | -0.725 | -1.020 | 1.769 | -2.763 | -0.227 | 0.854 | 0.419 | 0.384 | * -1.965 | -0.692 | * -2.361 | * -2.086 | -1.811 | -1.562 | -1.183 | -0.638 | -0.427 | * -2.047 | -1.413 |
| **R Caudate** | -0.875 | -3.238 | -1.400 | -16.880 | -2.038 | ** 2.772 | -0.752 | -1.111 | -0.763 | -0.520 | 0.347 | * -2.569 | -1.922 | ** -3.169 | -0.426 | * -2.047 | -1.124 | -1.426 | * -2.396 | -1.290 |
| **R Hippocampus** | -1.525 | -0.987 | -2.343 | -1.999 | -0.525 | 0.040 | 1.496 | 0.787 | 0.697 | -0.187 | 0.185 | * -1.976 | -0.561 | -1.798 | -0.333 | -1.242 | 0.344 | -1.406 | -0.603 | -0.557 |
| **R Pallidum** | -0.979 | -1.929 | -3.904 | 7.509 | 0.894 | -0.092 | 0.848 | 0.034 | -0.653 | 0.439 | -0.107 | -1.820 | -1.279 | ** -2.981 | -2.012 | * -2.129 | -1.404 | -1.727 | * -2.459 | -0.290 |
| **R Putamen** | 0.574 | -1.596 | 0.609 | -19.314 | 1.240 | 0.797 | 0.569 | -0.759 | -0.168 | -0.863 | 0.068 | ** -3.261 | -1.329 | *** -3.630 | ** -3.003 | * -2.470 | * -2.373 | -1.880 | -1.199 | -1.008 |
| **R Thalamus.Proper** | -5.134 | -7.549 | -6.213 | -14.981 | -0.151 | 1.152 | 1.165 | 0.474 | 0.180 | -1.273 | 0.098 | ** -2.962 | -1.812 | * -2.422 | -1.686 | ** -3.260 | -1.329 | * -2.147 | -1.590 | -1.279 |

Supplement Table 4 – Main analysis from Allegra MRI scanner only, uncorrected p-value. N = 2758
* - p < 0.05
** - p < 0.01
*** - p < 0.001

| Criteria | Number of Imaging Studies | Details |
| --- | --- | --- |
| All | 12,600 | Freesurfer analyses from T1 data collected on Allegra 3T and Skyra 3T MRI scanners |
| Successful Analyses | 10,120 | Successful Freesurfer analyses |
| Data in Range | 6,813 | Valid sex, valid project, only latest imaging study if subject has multiple MRI scans, and between date range |
| Exclude | 6,489 | Exclude QA, testing, phantom scans, motion artifacts, unknown/missing ages |
| Arbitrary cutoffs/Incomplete data | 6,176 | Freesurfer analyses that had any missing values, or values exceeding the arbitrary cutoffs, or did not having motion data |
| Healthy adults | 3,279 | Subjects explicitly marked as healthy controls or subjects enrolled in projects in which only healthy controls were enrolled, and between inclusive ages of 18 and 65 years. |

Supplement Table 5 – Criteria summary for data included in analysis

**Supplemental figures**

|  |  |
| --- | --- |

Supplement Figure 1 – MRI phantom signal to noise ratio (SNR) for available data for Allegra 3T and Skyra 3T MRI. Due to collection irregularities, fewer scans were performed during summer months.


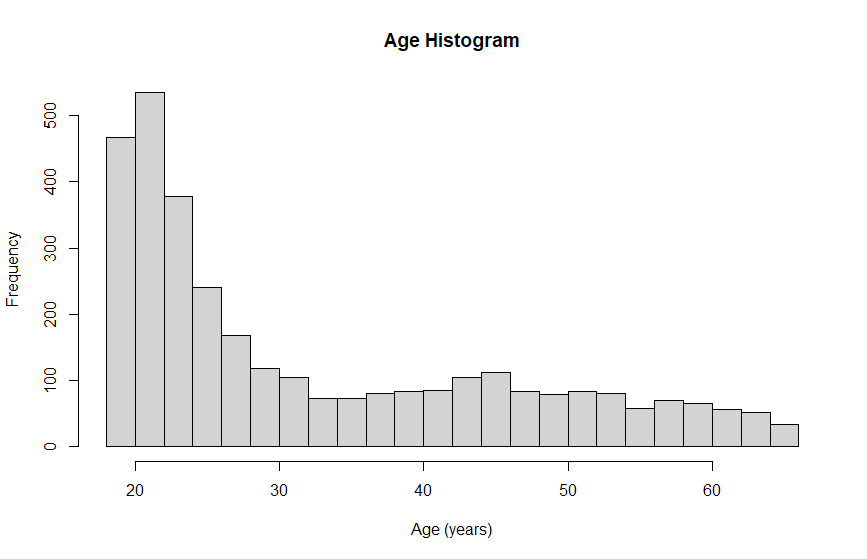

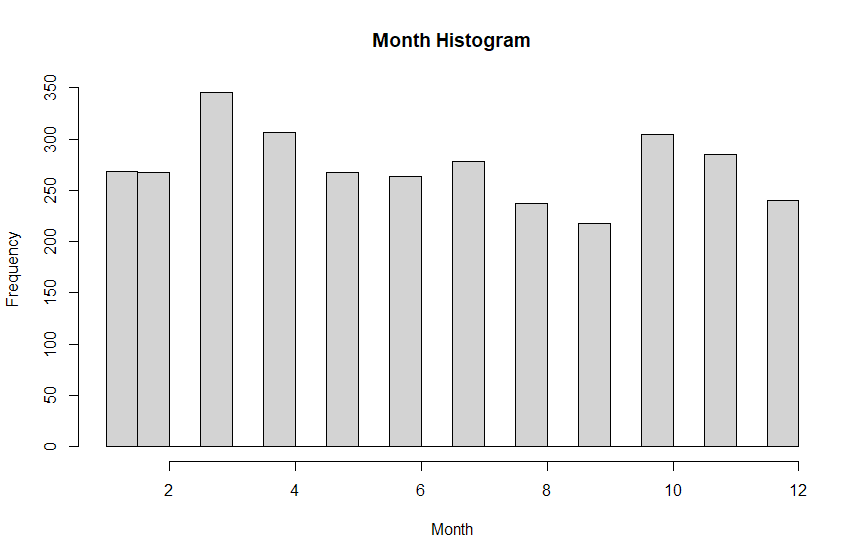

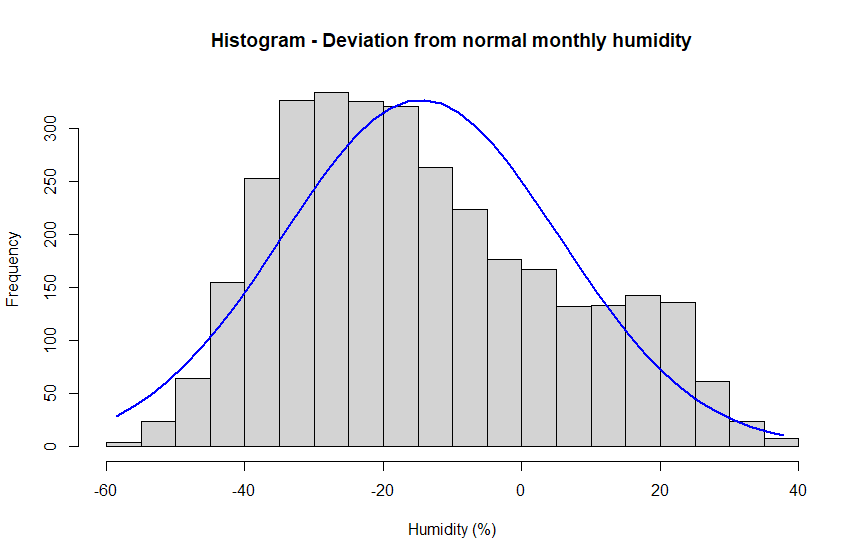

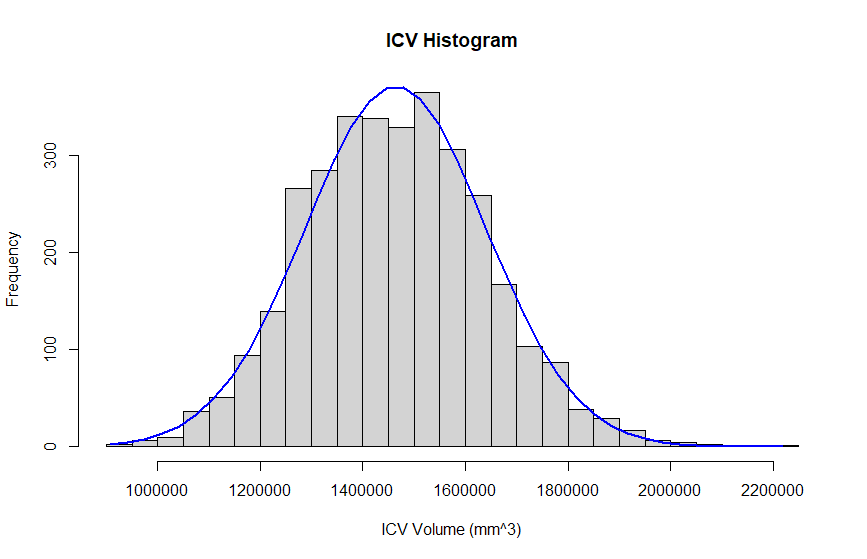

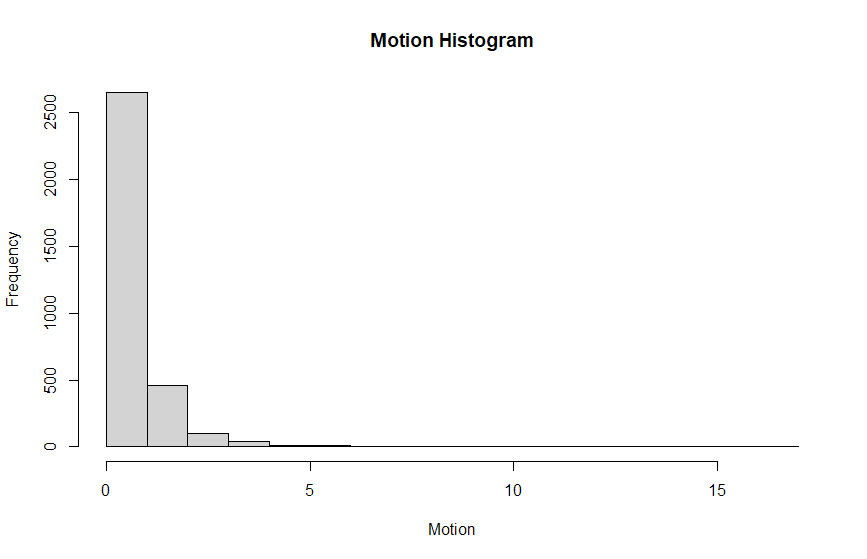

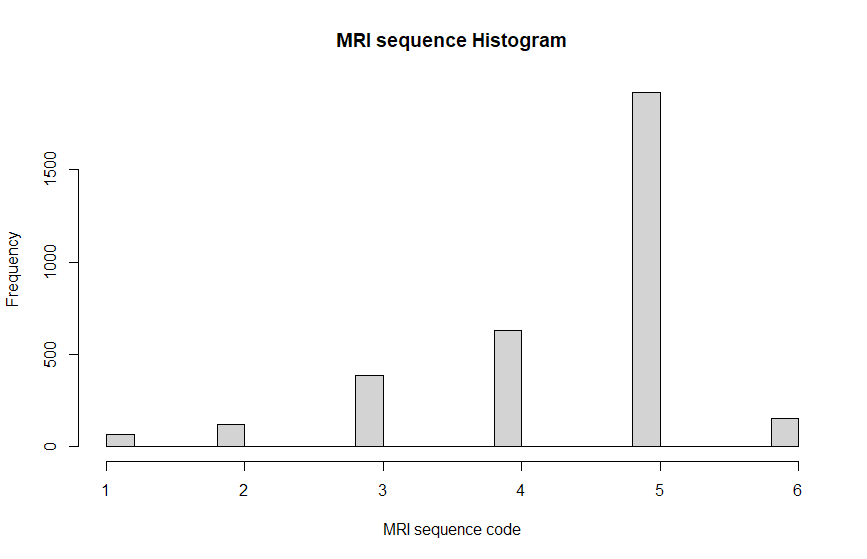

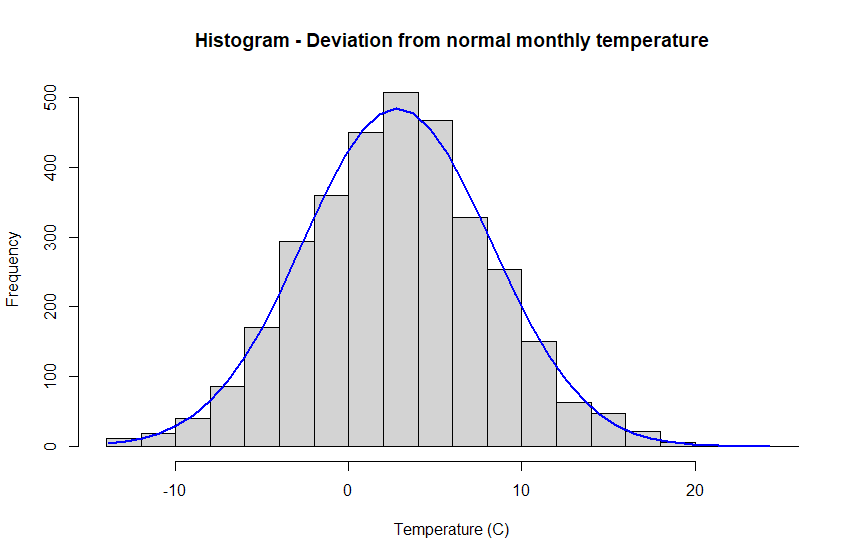

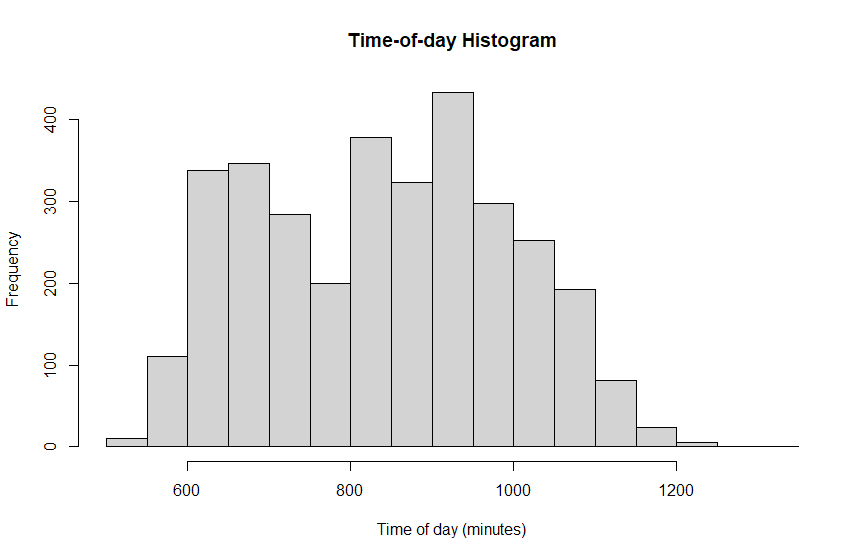


**QQ normality plots**

| 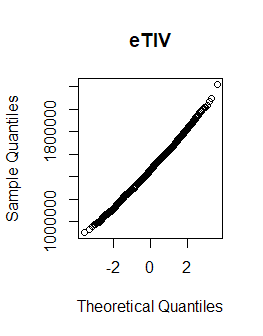 | 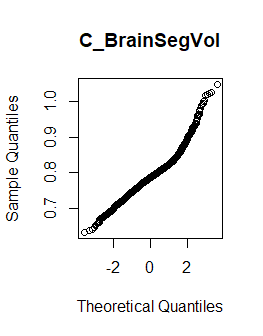 | 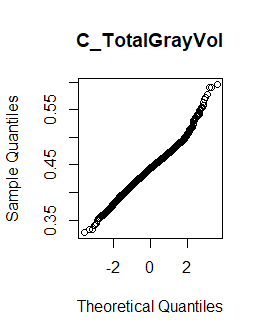 |
| --- | --- | --- |
| 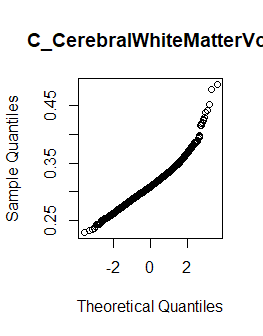 | 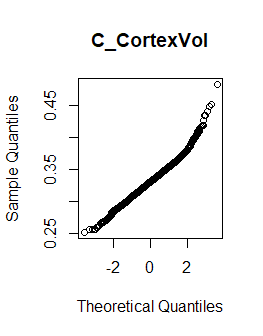 | 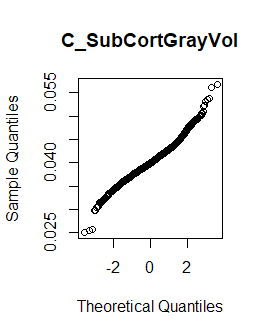 |
| 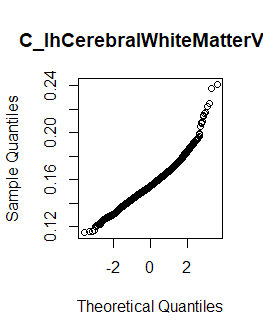 | 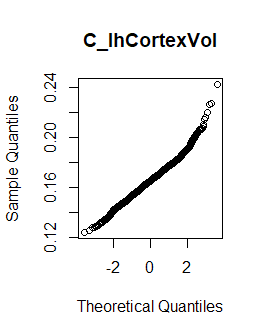 | 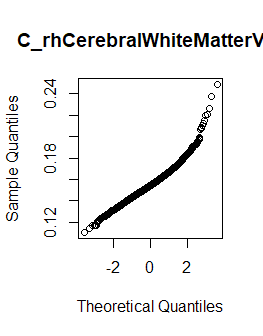 |
| 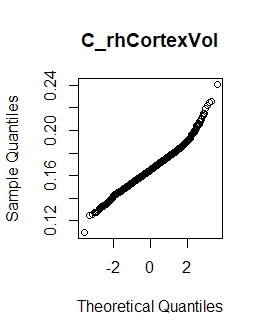 | 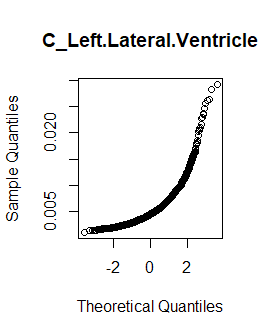 | 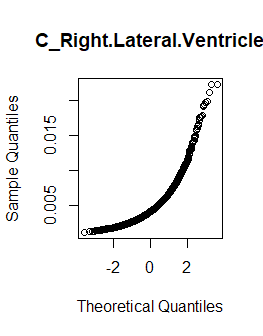 |
| 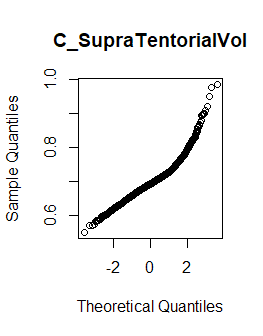 | 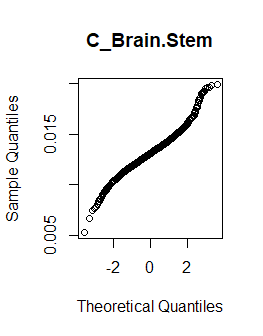 | 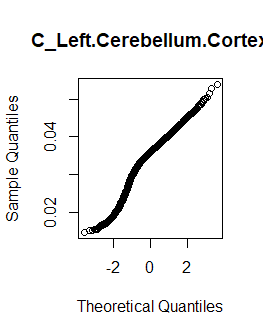 |
| 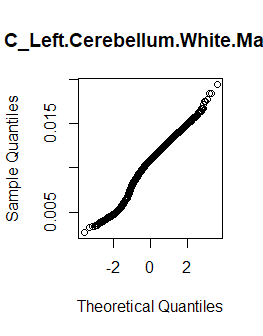 | 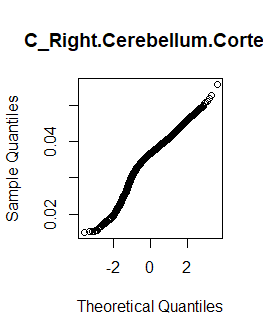 | 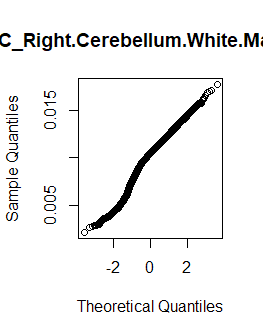 |
| 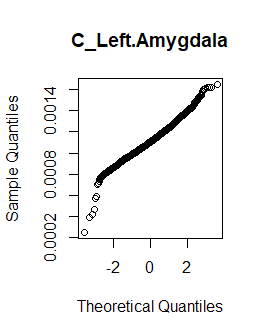 | 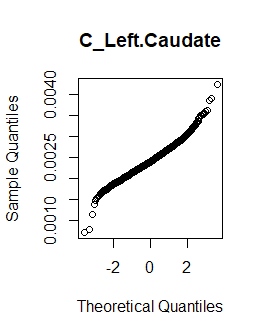 | 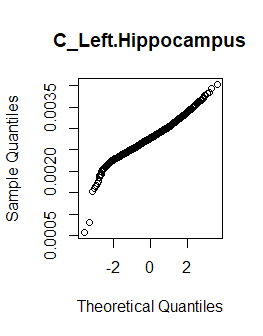 |
| 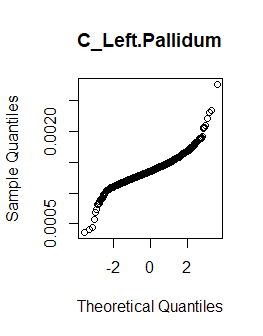 | 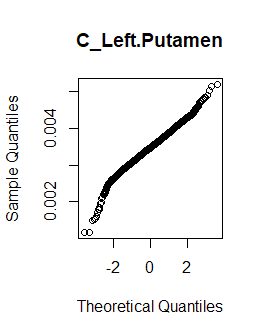 | 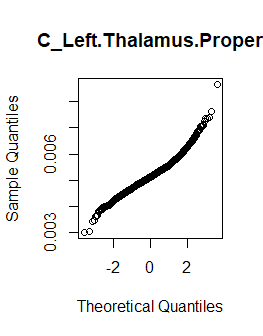 |
| 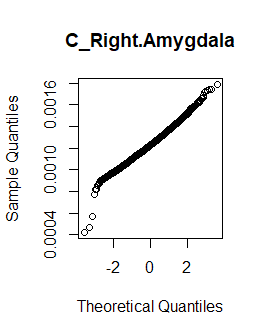 | 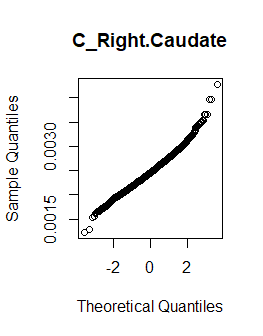 | 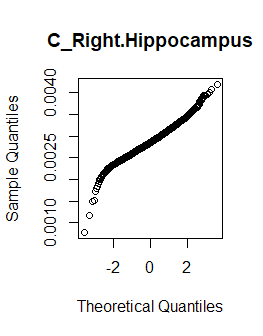 |
| 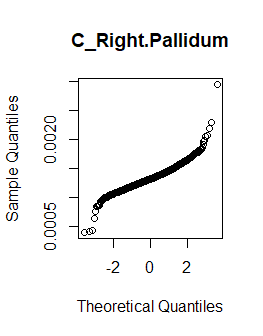 | 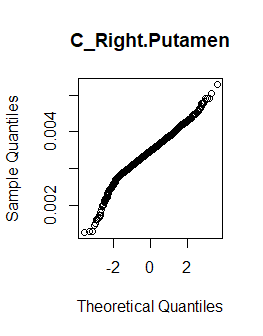 | 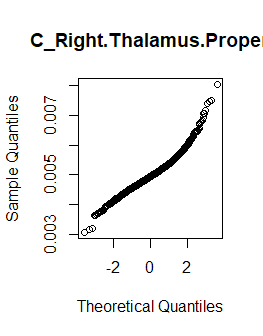 |
